# Electroanalytical Platform for Rapid *E. coli* O157:H7 Detection in Water Samples

**DOI:** 10.3390/bios14060298

**Published:** 2024-06-07

**Authors:** Kundan Kumar Mishra, Vikram Narayanan Dhamu, Chesna Jophy, Sriram Muthukumar, Shalini Prasad

**Affiliations:** 1 Department of Bioengineering, University of Texas at Dallas, Richardson, TX 75080, USA; 2 EnLiSense LLC, 1813 Audubon Pondway, Allen, TX 75013, USA

**Keywords:** electrochemical impedance spectroscopy, pathogens, *Escherichia coli*, immunosensor

## Abstract

There is a pressing need to enhance early detection methods of *E. coli* O157:H7 to mitigate the occurrence and consequences of pathogenic contamination and associated outbreaks. This study highlights the efficacy of a portable electrochemical sensing platform that operates without faradaic processes towards detecting and quantifying *E. coli* O157:H7. It is specifically tailored for quick identification in potable water. The assay processing time is approximately 5 min, addressing the need for swift and efficient pathogen detection. The sensing platform was constructed utilizing specific, monoclonal *E. coli* antibodies, based on single-capture, non-faradaic, electrochemical immunoassay principles. The *E. coli* sensor assay underwent testing over a wide concentration range, spanning from 10 to 10^5^ CFU/mL, and a limit of detection (LoD) of 1 CFU/mL was demonstrated. Significantly, the sensor’s performance remained consistent across studies, with both inter- and intra-study coefficients of variation consistently below 20%. To evaluate real-world feasibility, a comparative examination was performed between laboratory-based benchtop data and data obtained from the portable device. The proposed sensing platform exhibited remarkable sensitivity and selectivity, enabling the detection of minimal *E. coli* concentrations in potable water. This successful advancement positions it as a promising solution for prompt on-site detection, characterized by its portability and user-friendly operation. This study presents electrochemical-based sensors as significant contributors to ensuring food safety and public health. They play a crucial role in preventing the occurrence of epidemics and enhancing the supervision of water quality.

## 1. Introduction

*Escherichia coli*, colloquially referred to as *E. coli*, represents a group of bacteria characterized by their rod-shaped morphology and facultative anaerobic metabolism, categorized as Gram-negative organisms [1,2]. Among the diverse strains of *E. coli*, *E. coli* O157:H7 stands out for its propensity to colonize the human gastrointestinal tract, often precipitating severe health complications such as bloody diarrhea. Furthermore, it poses grave risks, including the onset of hemorrhagic colitis (HC) and hemolytic uremic syndrome (HUS) [1]. HC affects individuals across various age demographics, while HUS predominantly affects young children and the elderly, frequently culminating in acute renal failure and accounting for a significant portion of HUS cases in pediatric populations [1,2].

Estimates from the United States Department of Agriculture’s Economic Research Service (ERS) underscore the substantial economic impact of *E. coli*-related illnesses in the United States, exceeding USD 400 million annually [3]. The Centers for Disease Control and Prevention (CDC) further highlight the severity of the issue, reporting approximately 265,000 cases of Shiga toxin-producing *E. coli* (STEC) infections in the US every year, with *E. coli* O157 accounting for around 36% of these cases. Moreover, these infections result in an estimated 3600 hospitalizations and 30 fatalities annually in the United States [2,4,5]. Beyond the predominant *E. coli* O157:H7 strain, other serotypes of *E. coli*, collectively known as non-O157:H7 STECs, present significant community health-related issues because of their capacity to cause illness through diverse mechanisms. These serotypes, such as O26, O103, O111, O121, O45, and O145, have prompted regulatory agencies to implement stringent policies, particularly within the food industry. Detection of *E. coli* in water sources often indicates fecal contamination, while its presence in food samples may suggest the existence of enteropathogenic microbes, particularly in inadequately cooked or raw beef, unpasteurized milk, or juices [2,6,7,8,9]. These statistics and insights underscore the urgent need for effective measures to mitigate the risks posed by *E. coli* contamination and its potential impacts on public health and the economy.

In response to the challenges posed by *E. coli* contamination, various detection methods have been developed, leveraging immunological techniques integrated with nanotechnology to enhance sensitivity and specificity. These methods encompass nucleic acid-based electrochemical nanobiosensors and immunofluorescence strip sensors [10,11,12,13,14]. Furthermore, researchers have explored innovative modalities such as surface plasmon biosensors, which exploit the antibody–antigen reaction for signal generation, demonstrating promising detection thresholds for *E. coli* O157:H7 [12,15].

**Figure 1:**
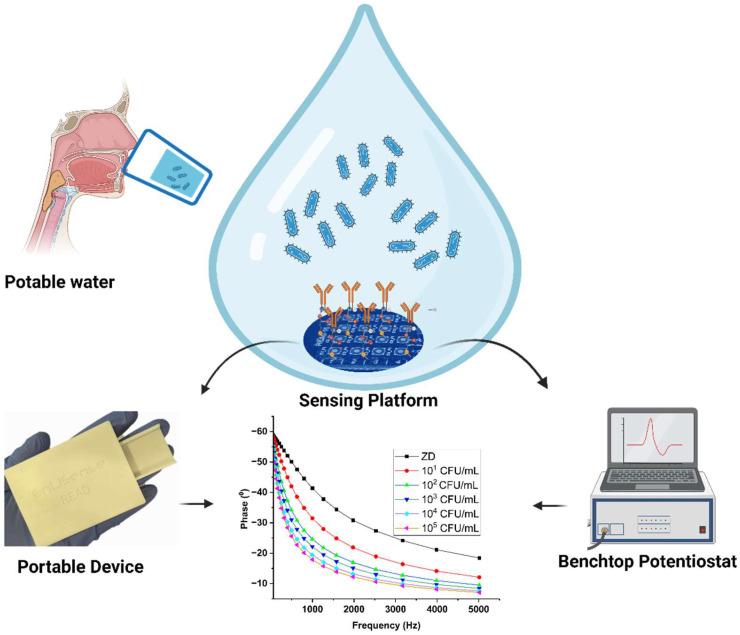
Schematic of the electrochemical detection of *E. coli* on the modified sensor platform, tested using both benchtop and portable devices.

The current state of the art in *E. coli* sensing predominantly relies on traditional methods such as culture-based ELISA and PCR techniques, which are often time-consuming and require specialized laboratory equipment. These methods involve isolating pathogen residues from water matrices, which can be cumbersome and may not be suitable for on-field deployment [16,17,18,19,20,21]. The gap lies in the need for a portable, label-free, electrochemical sensor capable of rapid and direct detection of *E. coli* in real field matrices. Existing methods lack the ability to provide timely results without the need for extensive preprocessing or laboratory facilities. Additionally, there is a demand for a sensor that maintains structural stability while achieving low limits of detection. Supplementary Table S1 presents a comparison of different immunosensors, revealing that label-free variants lack the sensitivity for direct *E. coli* detection. Our proposed platform endeavors to bridge this gap by combining the portability of electrochemical sensors with the direct detection capabilities essential for on-field deployment. It aims to overcome the limitations of traditional methods by offering timely results without extensive preprocessing or the requirement for specialized laboratory facilities [22,23]. Additionally, the proposed biosensor strives to maintain structural stability while achieving low limits of detection, ensuring its efficacy in real-world applications. Our study addresses this gap by developing an innovative electrochemical sensor tailored for on-field deployment, utilizing immobilized electrodes with specific antibodies and a crosslinker for direct pathogen detection in water. Through electrochemical impedance spectroscopy (EIS), the sensor efficiently transduces non-faradic signals generated by antigen–antibody interactions (as illustrated in Figure 1), presenting a portable and highly sensitive solution for on-field *E. coli* detection without the need for extensive preprocessing or laboratory equipment.

## 2. Materials and Methods

### 2.1. Materials and Reagents

The polyclonal *E. coli* O157:H7 antibodies were procured from Invitrogen (Gaithersburg, MD, USA), while heat-killed *E. coli* O157:H7 cells were purchased from Cedarlane (native antigen, Burlington, NC, USA). Phosphate-buffered saline (pH 7.4) and 3,3′dithiobis(sulfosuccinimidyl propionate were purchased from Thermo Fisher Scientific Inc.(Waltham, MA, USA) Potable water samples (Ozarka) were sourced from BlueTriton Brands Inc. (Dallas, TX, USA) and divided into multiple portions for storage at −20 °C until needed. Before experimentation, the diminutive samples underwent thawing to room temperature and subsequent centrifugation. High-purity chemicals were employed in this study, eliminating the necessity for further purification prior to utilization.

### 2.2. Sensor Fabrication

The sensor was carefully constructed on a small printed circuit board (PCB) platform, utilizing a two-electrode system. The working electrode comprises a gold electrode that was enhanced with a zinc oxide (ZnO) coating through electron vapor deposition techniques. The sensor platform, designed in the laboratory, is printed on a PCB-FR4 substrate, with an ENIG gold finishing on a copper clad board (Taichung, Taiwan). It includes an array of 16 electrodes, each meticulously surrounded by a gold reference electrode, ensuring the stability and reliability of its performance.

### 2.3. ATR-IR Spectroscopy Setup

The Nicolet iS-50 FTIR instrument by Thermo Scientific Inc. was used to capture the infrared spectra (IR) in “Attenuated Total Reflectance” mode. The detector material, deuterated triglycine sulfate (DTGS), and a KBr window were utilized. Spectrum acquisition employed a germanium crystal, providing a resolution of 4 cm^−1^ in the wavelength range of 4000 cm^−1^ to 400 cm^−1^, with 256 scans. ZnO deposition on glass substrates followed the same parameters as the sensor substrate deposition. DTSSP was used for functionalizing and immobilizing the sensor surface, similar to the sensor immunoassay described in Section 2.5. *E. coli* antibodies were then individually incubated on the sensor surfaces in 100% PBS post-functionalization.

### 2.4. Electrochemical Studies

In this study, we evaluated the sensor’s response using electrochemical impedance spectroscopy (EIS). Using a minimal input voltage, i.e., 10 mV over a frequency span ranging from 1000 to 80 Hz, we analyzed the impedance response to capture subtle variations arising from the interaction between antibodies and antigens. Furthermore, we utilized Bode and Nyquist plots for a comprehensive analysis of the sensor’s behavior. These plots offer valuable insights into the frequency-dependent impedance response and provide a visual representation of the electrochemical system’s complex plane. Through the examination of the Bode and Nyquist plots, we gained a deeper understanding of the sensor’s performance characteristics, facilitating a more thorough evaluation of its functionality and potential applications.

### 2.5. Modification of Sensing Platform

The process of modifying the electrode surface involved several meticulously executed stages. Initially, the sensor surface underwent thorough cleansing with phosphate-buffered saline (PBS). Subsequently, a mixture containing DTSSP (10 mM) and antibodies (10 mg/mL) was carefully applied onto the surface, where it was allowed to incubate under controlled conditions at a temperature of 4 °C for 30 min. After this period of incubation, the sensor underwent another meticulous washing step with PBS to ensure the removal of any excess or unbound reagents. Following the completion of the washing step, 5 microliters of the actual sample under investigation were precisely applied onto the sensor surface. This sample was then left to incubate for an additional 5 min, allowing for any potential interactions between the sample components and the modified sensor surface to occur. Upon completion of this incubation period, the data collection process commenced utilizing electrochemical impedance spectroscopy (EIS), a sophisticated technique capable of providing detailed insights into the electrochemical characteristics of the sensor under various conditions.

### 2.6. Statistical Analysis

The data provided include mean values along with their corresponding standard error of the mean (SEM).

The data underwent analysis using GraphPad Prism (version 8.01), a software engineered by GraphPad Software in La Jolla, CA, USA. Further analyses were carried out using Origin 2023. Reaction mechanism graphics were created using BioRender (BioRender.com), while ZView software (Version: 3.5g) was employed for data modeling and the equivalent circuit study.

## 3. Results and Discussion

### 3.1. Sensor Characterization

**Figure 2:**
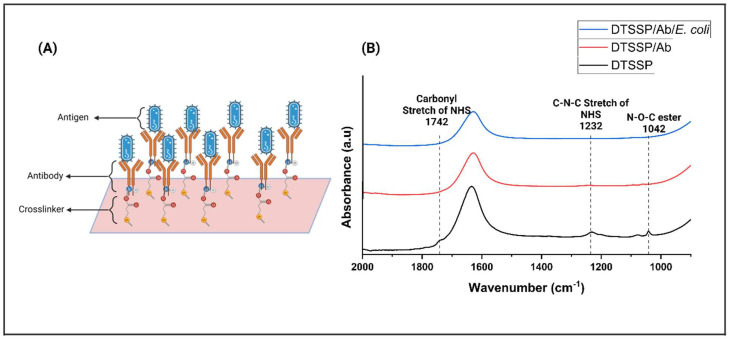
(**A**) Schematic representation of the immobilizing linker molecules on the substrate of the sensor and covalently conjugating the antibody to the linker. (**B**) FTIR spectra of (i) DTSSP (black spectrum) functionalized on the ZnO surface, (ii) conjugation of *E. coli* Ab and DTSSP (red spectrum), and (iii) conjugation of *E. coli* antigen on the surface of the Ab (blue spectrum).

Affinity-based functionalization was employed as a pivotal strategy for the detection of *E. coli*. The initial step involved the thiol-based crosslinker, DTSSP, attaching to the sensor surface, followed by fixing the *E. coli* antibody onto the DTSSP-coated surface, depicted in Figure 2A.

To confirm the effective functionalization of the sensor, atomic force microscopy (AFM) and Fourier-transform infrared spectroscopy (FTIR) were employed. A ZnO film was carefully applied onto the sensor surface via vapor deposition to optimize surface properties and increase sensitivity to target pathogens. ZnO contributes to improved surface interactions, thereby enhancing the sensor’s ability to effectively detect pathogens. AFM topographic images (Supplementary Figure S2A,B) show the sensor surface both before and after testing with *E. coli* bacteria. These images revealed the presence of antibodies, measuring 300–450 nm, adhered to the crosslinker on the sensor platform. After testing with *E. coli*, the size of the *E. coli* cells (1.5 to 2 µm) confirmed their presence on the sensor surface. FTIR analysis was performed to confirm the presence of *E. coli* and *E. coli* antibodies on the sensor platform. The acquired FTIR spectra of the synthesized sensor provided compelling evidence of the presence of the antibody on the functionalized sensor platform. Remarkably, a robust peak at 1742 cm^−1^ was prominently observed upon the surface modification with DTSSP [24]. Figure 2B vividly depicts the C–N–C stretch of the N-hydroxysuccinimide (NHS) at 1232 cm^−1^ and the N–O–C esters peak at 1042 cm^−1^. Subsequently, the vanishing of these peaks at 1742 cm^−1^, 1232 cm^−1^, and 1042 cm^−1^ confirmed the successful immobilization of the antibody onto the ZnO surface, facilitated by DTSSP through the formation of an amide bond [25,26,27]. Following the modification of the sensor with the antibody, *E. coli* pathogens were meticulously introduced onto the sensor surface. Intriguingly, the intensity of the peak was observed to diminish significantly when the *E. coli* bound to its corresponding antibody [27]. This compelling observation provided further validation of the functionality and efficacy of the sensor in the accurate and sensitive detection of *E. coli* pathogens, thereby affirming its potential utility in practical applications.

### 3.2. Electrochemical Signal Response on the Modified Sensor Platform

Electrochemical impedance spectroscopy (EIS) was utilized to evaluate the electrochemical functionality of a customized sensor tailored for *E. coli* pathogen detection [28]. This modification enabled the utilization of electrochemical measurements, particularly through EIS, employing a small applied 10 mV AC bias [24,28]. The changes in the observed signal were a result of the adjustment of the electrical charge within the double layer on the sensor surface, resulting from the attachment of the target antigen to the corresponding antibodies immobilized on the sensor surface. This intricate process facilitated the accurate and sensitive detection of *E. coli* pathogens, enhancing the sensor’s functionality for real-world use in diverse fields like medical diagnosis and environmental surveillance. These changes in impedance were used to generate calibrated dose–response (CDR) plots corresponding to variations in antigen concentrations. In the CDR measurements, specific spiked doses of the *E. coli* antigen were individually prepared through serial dilution, ranging from zero (control matrix) to concentrations of 10^1^, 10^2^, 10^3^, 10^4^, and 10^5^ CFU/mL. These measurements were conducted in potable water matrices to comprehensively evaluate the sensor’s response across a range of concentrations in potable water.

**Figure 3:**
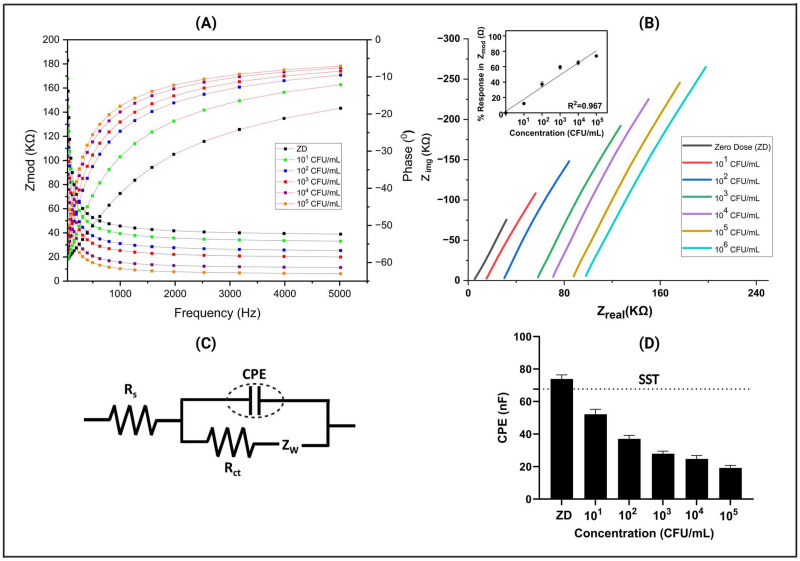
(**A**) Characteristic Bode response plot for the CDR study showcasing the behavior of *E. coli* antigens on the sensor platform across spiked dose concentrations (ZD) ranging from 10^1^ CFU/mL to 10^5^ CFU/mL in the potable water matrix. (**B**) Nyquist plot of EIS responses depicting the sensor’s reaction to different concentrations of *E. coli.* (**C**) Randles equivalent circuits corresponding to the sensor platform. (**D**) Variation in electrical double-layer capacitance with different concentrations of *E. coli* spiked in potable water.

In Figure 3A, a detailed analysis of the dose-dependent Bode plot, obtained through EIS measurements, is presented. This plot showcases the variation in impedance (Zmod, measured in Ω) in relation to frequency (measured in Hz) for potable water spiked with different concentrations of *E. coli*. The impedance values ranged from 7950 Ω to 41,000 Ω for the zero dose (control) and increased with higher concentrations of *E. coli* doses. This linear correlation between impedance and antigen concentrations suggests an amplification in antigen binding, leading to the modulation of the double-layer charge and subsequent signal fluctuation across a wide frequency spectrum [29,30]. Notably, the peak signal-to-noise ratio was observed at 200 Hz, prompting its selection as the optimal frequency for analyzing and generating the calibrated dose–response plot. This discovery offers significant understanding regarding the detection sensitivity and dynamic range of the sensing platform, enhancing its potential for real-world use in diverse fields like medical diagnosis and environmental surveillance.

Furthermore, in the inset Nyquist plot illustrating Z_img_ versus Z_real_ (shown in Figure 3B) for the same measurement, we observed a significant trend indicating a decline in imaginary impedance as antigen concentration increased. This transition towards more real values is highly suggestive of antigen binding, a pivotal event in our immunoassay. It is important to note that this phenomenon is attributable to a non-faradaic process devoid of redox material, which underscores the specificity of our assay for antigen detection. Furthermore, this shift in impedance characteristics signifies an inverse relationship between the imaginary part of impedance and the electrical double-layer capacitance, providing valuable insights into the underlying mechanisms at play [25].

Moreover, in our comprehensive analysis, we also observed a shift in the Z_real_ value when the concentration increased. These shifts represent the Rs (solution resistance) value, indicating alterations in the bulk solution properties, possibly influenced by changes in antigen concentration. This Rs shift suggests dynamic changes in the electrochemical environment, potentially reflecting variations in the ionic conductivity of the solution due to antigen–antibody interactions. To delve deeper into the electrochemical behavior, we employed the Randles equivalent circuit (shown in Figure 3C). The Rs parameter signifies the total resistance of the bulk solution, which was arranged in series with a parallel configuration comprising zinc oxide resistance (RZnO) and zinc oxide capacitance (CZnO). This arrangement was further connected in series with another parallel RC circuit, specifically at lower frequencies. Within this RC circuit, two components are notable as the following: the charge transfer resistance (Rct), which characterizes the resistance encountered during the transfer of charge at the electrode–electrolyte interface, and the constant phase element (CPE). The presence of the CPE suggests a non-ideal behavior in the system, indicating complexities such as surface roughness, double-layer capacitance, or other phenomena deviating from pure capacitance. This comprehensive model, including the Randles equivalent circuit and its components, is widely recognized in the field of impedance spectroscopy in electrochemical systems [31]. By extracting the data related to the constant phase element (CPE) from the Randles equivalent circuit, we uncovered significant variations in the CPE values corresponding to different antigen concentrations. This intriguing finding underscores the dynamic nature of the interaction between the antigen and antibody within our system.

In Figure 3D, a representative calibration dose response (CDR) plot is depicted, featuring a specific signal threshold (SST) determined to differentiate between a signal and noise. This threshold was calculated as three times the standard deviation (SD) of the blank, added to the mean of the blank concentration (ZD) [25]. Notably, as the antigen concentration increases, a discernible alteration in the constant phase element (CPE) becomes apparent. These pronounced changes in CPE values indicate a significant modification in the electrical properties of the interface. This alteration is directly associated with the binding events occurring between the biomolecules, reflecting the dynamic interactions between antigens and antibodies. Hence, our comprehensive analysis not only validated the effectiveness of our immunoassay in detecting antigen presence but also provided crucial insights into the underlying electrochemical processes governing the antigen–antibody interaction. This detailed version provides a structured and comprehensive explanation of the observations made in the Nyquist plot, the implications of the shift in impedance characteristics, the significance of the Rs value shift, the utilization of the Randles equivalent circuit, and the insights gained from the CPE analysis.

### 3.3. Spike and Recovery Study for E. coli

**Figure 4:**
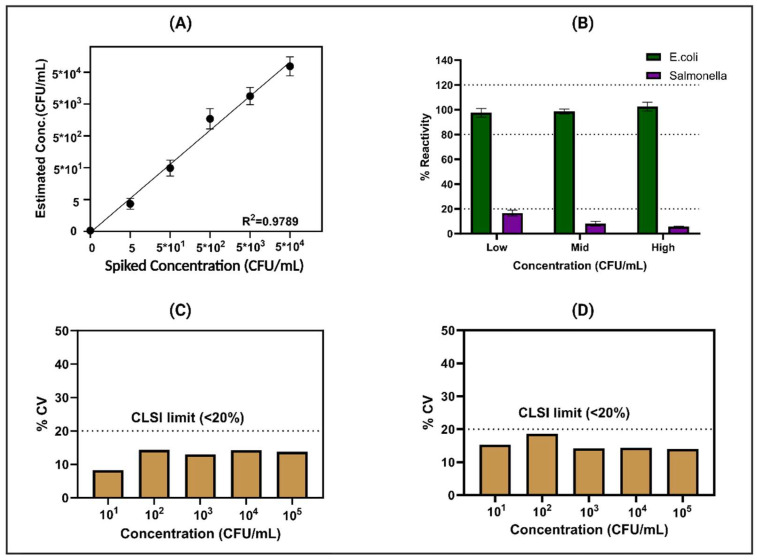
(**A**) Signal response of the sensor platform to spiked dose concentrations ranging from 5 CFU/mL to 5 × 10^4^ CFU/mL in potable water. (**B**) Results of the specificity study, showcasing the sensor response to increasing concentrations of *E. coli* compared to *Salmonella* concentration (low (10^3^ CFU/mL), mid (10^5^ CFU/mL), high (10^7^ CFU/mL)). The reproducibility and repeatability of the sensor platform were assessed through statistical analysis, as depicted in (**C**) for intra-assay variation (N = 8) and (**D**) for inter-assay variation (n = 3), showing consistent performance confirmed by %CV.

Spike and recovery methodology is a common analytical technique used to assess the accuracy and reliability of a measurement method. In this method, we spiked a known concentration of *E. coli* to the sample and calculated the percentage recovery based on the CDR obtained (shown in Supplementary Figure S1). The goal was to evaluate the precision and bias of the analytical procedure by comparing the expected concentration (based on the spike) with the measured concentration. During the spike and recovery experiments, *E. coli* was deliberately introduced at random concentrations spanning from the control or zero dose (ZD) to 5, 5 × 10^1^, 5 × 10^2^, 5 × 10^3^, and 5 × 10^4^ CFU/mL, and recovery was calculated based on the CDR obtained in potable water sources shown in Supplementary Figure S1. The study encompassed N = 4 replicates, with Figure 4A illustrating the %recovery plot in potable water, exhibiting a robust correlation with an R^2^ value of 0.97. Throughout the specified concentration range, the percentage error consistently remained below 20%, indicating a close correspondence between the developed sensor’s readings and the actual *E. coli* concentrations in the potable water. Moreover, the sensor’s limit of detection (LoD) was established at 1 CFU/mL, determined through the signal-to-noise (S/N) threshold method. This determination underscores the sensor’s ability to detect even minute levels of *E. coli* contamination [25].

### 3.4. Cross-Reactivity Study

Ensuring selective performance, especially in real-time applications, is imperative for a sensor platform’s effectiveness. In practical scenarios, the matrix and electrode encounter diverse nutrients, which can influence mineral and nutrient binding. Moreover, data interpretation may be affected by the presence of structurally similar pathogens in similar or higher concentrations. Our comprehensive cross-reactivity testing, as depicted in Figure 4B, involved the evaluation of the modified sensor platform’s response to cross-reactive antigens spiked at various concentrations alongside the analytes of interest in potable water. The response plot, specifically focused on *E. coli* detection within the water matrix, is vividly illustrated in Figure 4B. Notably, this plot demonstrates a discernible 16% alteration in total impedance subsequent to exposure to high doses of *Salmonella* on the *E. coli*-Ab sensor platform. Conversely, minimal changes of less than 10% were observed following exposure to medium and low doses. These results highlight the sensor platform’s remarkable level of specificity, indicating minimal cross-reactivity across varying concentrations of structurally similar pathogens. Such selectivity is paramount for ensuring the dependable detection of pathogens in real-world water samples, where the presence of diverse substances and potential contaminants can potentially complicate analytical processes [32]. The ability to distinguish between analytes of interest and cross-reactive antigens enhances the sensor platform’s utility and reliability in practical applications, emphasizing its potential for field deployment under varied environmental conditions.

### 3.5. Reproducibility and Repeatability

The sensor platform’s capability to effectively detect low concentrations of *E. coli* in potable water has been substantiated through rigorous testing. In this study, comprehensive evaluations were conducted to thoroughly assess both the intra-assay repeatability and inter-assay reproducibility of the system. Illustrated in Figure 4C are the results depicting the coefficient of variation (%CV) across a spectrum of *E. coli* concentrations, ranging from 10^1^ to 10^5^ CFU/mL. Remarkably, the data showcase that the sensors consistently exhibited a coefficient of variation (CV) of less than 20% across the various concentrations tested. This remarkable consistency suggests that intra-assay variability adheres closely to the stringent guidelines established by the Clinical and Laboratory Standards Institute (CLSI) [33], underscoring the precision and reliability of the sensor platform’s performance. Moreover, Figure 4D presents the inter-assay variation, which was meticulously conducted across a sample size of n = 3 sensors. Once again, the findings demonstrate a coefficient of variation below 20% across different concentrations of *E. coli*. This adherence to CLSI guidelines further validates the robustness and dependability of the sensor platform in accurately detecting *E. coli* in water samples. These compelling results collectively affirm the consistent and reliable performance of both sensors, highlighting their suitability for real-world applications where precise and accurate detection of *E. coli* is paramount for safeguarding public health and ensuring water quality.

### 3.6. Comparative Analysis of Benchtop and Portable Device Measurements

**Figure 5:**
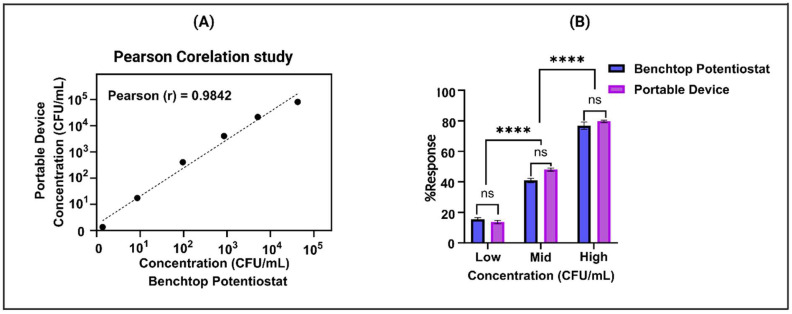
(**A**) Analysis of correlation between portable and benchtop devices at five concentrations showing linear fit. (**B**) Comparison of doses measured by portable and benchtop devices: paired *t*-test results. (ns represent no statistically significant difference between the corresponding doses, **** represent significant difference between the low to mid concentration and mid to high concentration readings for both devices).

After successfully evaluating the sensor platform’s performance in detecting *E. coli* in potable water, we proceeded to assess its potential transition to a portable device. This entailed conducting a trial to assess the viability of deploying the sensor under real-world conditions. To assess the functionality of the portable device, alongside the laboratory potentiostat acting as a benchmark, an extensive Pearson correlation analysis was performed. To confirm the effectiveness of the portable device, a rigorous parametric correlation test utilizing linear alignment was carried out to compare the acquired results with those obtained from the laboratory equipment. The portable device demonstrated a robust correlation with data from the reference instrument, showing a Pearson correlation coefficient (Pearson r) of 0.9842, illustrated in Figure 5A. Moreover, intriguing insights emerged from the comparison between the benchtop potentiostat and the portable device, meticulously depicted in Figure 5B. Through the execution of a paired *t*-test, it was discerned that there existed no statistically significant difference between the corresponding doses when measured by both instruments, underscored by a calculated *p*-value of 0.4024. This indicates that the instruments yielded similar results for identical doses, suggesting a level of consistency and reliability in their measurements. However, it was observed that there was a significant difference between the low to mid concentration and mid to high concentration readings for both the benchtop potentiostat and the portable device, with a *p*-value of 0.0001. This suggests that while the instruments yield similar results for identical doses, they both provide consistent and unbiased predictions of adulteration levels. This consistency is crucial for ensuring that either instrument can be reliably used for accurate assessments in various applications.

## 4. Conclusions

This study showcases the effectiveness of electrochemical impedance spectroscopy (EIS) in swiftly and reliably detecting *E. coli* in potable water. Through the integration of immunoassays and antibodies, the sensor’s selectivity is enhanced, allowing for precise identification of target antigens in potable water. Our system enables rapid detection, providing results in less than 5 min with a minimal sample volume requirement of only 5 µL. Achieving an impressive limit of detection (LoD) for *E. coli* at 1 CFU/mL, the adapted sensor demonstrates consistent performance across both laboratory-based testing and portable device applications, effectively covering a wide range of pathogen concentrations from 10^1^ to 10^5^ CFU/mL. Rigorous assessment of cross-reactivity with *Salmonella* has been conducted on the sensor platform. The fruition of this electrochemical-based sensing platform signifies a promising avenue for detecting pathogens in water samples. Through its portable and user-friendly design, our approach holds considerable potential for field-deployable monitoring, thereby enabling the prompt identification of pathogen contamination in potable water. These research results are fundamental for advancing electrochemical sensing platforms, making significant contributions to mitigating bacterial outbreaks, and upholding the sanitation standards of potable water. By addressing crucial provocation in *E. coli* sensing, these findings are pivotal for ensuring public hygiene and wellness.

## Data Availability

Data supporting the reported results are contained within the article and Supplementary Materials. No new data were created beyond those presented in the study.

## Supplementary Materials

The following supporting information can be downloaded at: https://www.mdpi.com/article/10.3390/bios14060298/s1, Figure S1: A calibration dose–response study was performed on the sensor platform for *E. coli* O157:H7 in potable water, covering spiked dose concentrations ranging from ZD to 10^5^ CFU/mL; Figure S2: (A) Atomic force microscopy (AFM) images showing the presence of antibodies (300–450 nm) on the sensor platform. (B) Sensor surface after exposure to *E. coli*, with *E. coli* bacteria measuring 1.5 to 2 µm, confirming their presence on the sensor surface; Table S1: Comparison of the developed immunosensor with other, studied, label-free detection methods of *E. coli* 0157:H7. References [34,35,36,37,38,39,40,41,42] are cited in Supplementary Materials.
