# Supplementary material for: Electroanalytical Platform for Rapid E. coli O157:H7 Detection in Water Samples
Source: Biosensors (Basel). 2024 Jun 7;14(6):298. doi: 10.3390/bios14060298 (PMC11201563; doi:10.3390/bios14060298)
Supplement: Supplementary file 1 [file biosensors-14-00298-s001.zip › biosensors-3015113-supplementary.pdf]

Supplementary Materials

Electroanalytical Platform for Rapid *E. coli* O157:H7 Detection in Water Samples

Kundan Kumar Mishra[1], Vikram Narayanan Dhamu[2], Chesna Jophy[1],
Sriram Muthukumar[2], Shalini Prasad [1,2*]
[1]Department of Bioengineering, University of Texas at Dallas, Richardson, TX 75080, USA,
[2]EnLiSense LLC, 1813 Audubon Pondway, Allen, TX 75013, USA.
Corresponding email: shalini.prasad@utdallas.edu

Table S1. Comparison of the developed immunosensor with other, studied, label-free detection methods of *E. coli* 0157:H7.

Immunosensor/ Device	Sample Type	LoD (CFU/mL)	Linear Range (CFU/mL)	Assay Time (min)	Reproducibility	Sensitivity	Selectivity (Cross reactivity)	Ref.
DPV	Licorice extract	80	$5*10^2$–$5*10^7$	150	RSD <5%	0.368 mA/CFU/mL	–	[1]
CV–DPV	Water	17	$1.7*10^1$–$1.1*10^7$	35	–	–	<20% against *Salmonella typhimurium*	[2]
DPV	Spring water and milk	34	$7.8*10^1$–$7.8*10^6$	150	RSD <3.4%	–	<15% against *Salmonella*, *E. coli* DH5α, and *E. coli* O149	[3]
DPV-EIS-CV	Water	2	$2.1*10^1$–$2.1*10^7$	20	RSD = 6.01%	–	20% and 30 % against *Salmonella typhimurium* and *Pseudomonas aeruginosa*	[4]
CV	Urine and water	4	4–10^6	70	%CV = 3.2 % and 6.3 %	353.17 mA/CFU/mL	<20% against *Proteus mirabilis*, *Enterococcus faecalis*, *Staphylococcus haemolyticus*, and *Pseudomonas aeruginosa*	[5]
EIS	Drinking water	75	$1.0*10^2$–$1.0*10^5$	70	RSD = 4.8%	6094 mA/CFU/mL	24.3 % and 17.9% against *E. coli* DH 5 and Gram-positive *M. luteus*	[6]
CV–CA	Beef and water	309	10^2–10^5	70–90	%CV = 6.7%	–	<25% against *Salmonella typhimurium*	[7]
CV	Ground beef	52	10^1–10^6	60	–	18.80 mA/CFU/mL	<17% against *Salmonella typhimurium* and *E. coli* K12	[8]
CV	PBS buffer	30	$3*10^1$–$3*10^7$	–	RSD = 8.9%	2.78 mA/CFU/mL	<15% against *E. coli* O124, *P. aeruginosa*, and *S. enteritidis*	[9]
EIS	Potable water	1	10–10^5	5	%CV <20% intra-assay variation and inter-assay variation	1453 Ω/CFU/mL	10–16% against *Salmonella typhimurium*	This work

DVP—Differential pulse voltammetry; CV—cyclic voltammetry; CA—chronoamperometry; EIS—electrochemical impedance spectroscopy; RSD—relative standard deviation; CV—coefficient of variation.

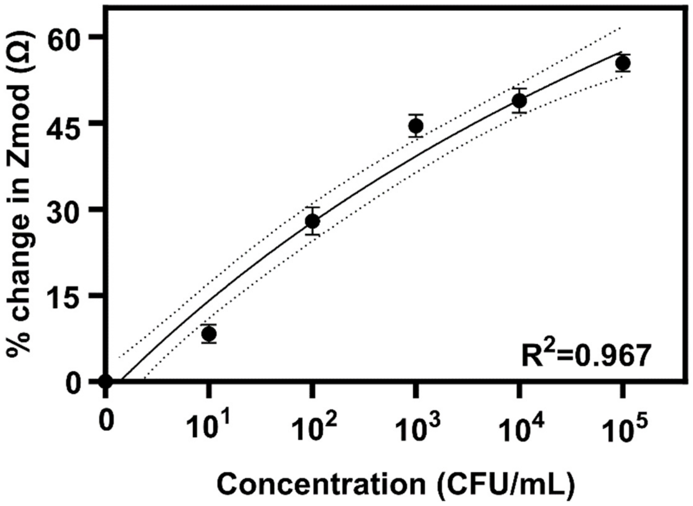

Figure S1. A calibration dose–response study was performed on the sensor platform for *E. coli O157:H7* in potable water, covering spiked dose concentrations ranging from ZD to 10^5 CFU/mL.

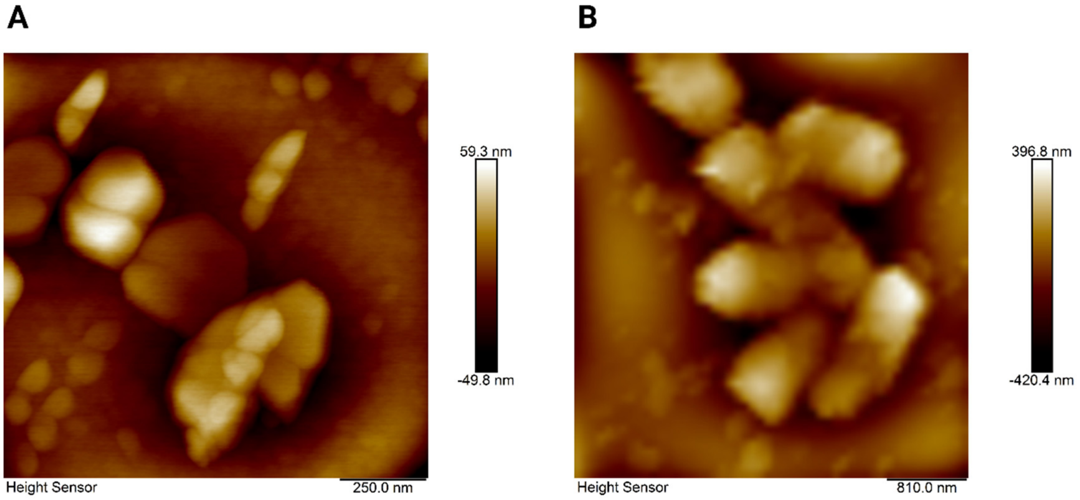

Figure S2. (A) Atomic force microscopy (AFM) images showing the presence of antibodies (300–450 nm) on the sensor platform. (B) Sensor surface after exposure to *E. coli*, with *E. coli* bacteria measuring 1.5 to 2 µm, confirming their presence on the sensor surface.

References

1. Wang, H.; Zhao, Y.; Bie, S.; Suo, T.; Jia, G.; Liu, B.; Ye, R.; Li, Z. Development of an Electrochemical Biosensor for Rapid and Effective Detection of Pathogenic Escherichia Coli in Licorice Extract. *Applied Sciences (Switzerland)* **2019**, *9*, doi:10.3390/app9020295.
2. Housaindokht, M.R.; Sheikhzadeh, E.; Pordeli, P.; Rouhbakhsh Zaeri, Z.; Janati-Fard, F.; Nosrati M. Mashreghi, M.; Nakhaeipour, A.; A. Esmaeili, A.; Solimani, S. A Sensitive Electrochemical Aptasensor Based on Single Wall Carbon Nanotube Modified Screen Printed Electrode for Detection of Escherichia Coli O157:H7. *Adv Mater Lett* **2018**, *9*, 369–374, doi:10.5185/amlett.2018.1701.
3. Guo, Y.; Wang, Y.; Liu, S.; Yu, J.; Wang, H.; Cui, M.; Huang, J. Electrochemical Immunosensor Assay (EIA) for Sensitive Detection of E. Coli O157:H7 with Signal Amplification on a SG-PEDOT-AuNPs Electrode Interface. *Analyst* **2015**, *140*, 551–559, doi:10.1039/c4an01463d.
4. Shahrokhian, S.; Ranjbar, S. Aptamer Immobilization on Amino-Functionalized Metal-Organic Frameworks:

An Ultrasensitive Platform for the Electrochemical Diagnostic of: Escherichia Coli O157:H7. *Analyst* **2018**, *143*, 3191–3201, doi:10.1039/c8an00725j.
5. Shoaie, N.; Forouzandeh, M.; Omidfar, K. Voltammetric Determination of the Escherichia Coli DNA Using a Screen-Printed Carbon Electrode Modified with Polyaniline and Gold Nanoparticles. *Microchimica Acta* **2018**, *185*, doi:10.1007/s00604-018-2749-y.
6. Yang, H.; Zhou, H.; Hao, H.; Gong, Q.; Nie, K. Detection of Escherichia Coli with a Label-Free Impedimetric Biosensor Based on Lectin Functionalized Mixed Self-Assembled Monolayer. *Sens Actuators B Chem* **2016**, *229*, 297–304, doi:10.1016/j.snb.2015.08.034.
7. Hassan, A.R.H.A.A.; de la Escosura-Muñiz, A.; Merkoçi, A. Highly Sensitive and Rapid Determination of Escherichia Coli O157: H7 in Minced Beef and Water Using Electrocatalytic Gold Nanoparticle Tags. *Biosens Bioelectron* **2015**, *67*, 511–515, doi:10.1016/j.bios.2014.09.019.
8. Xu, M.; Wang, R.; Li, Y. An Electrochemical Biosensor for Rapid Detection of: E. Coli O157:H7 with Highly Efficient Bi-Functional Glucose Oxidase-Polydopamine Nanocomposites and Prussian Blue Modified Screen-Printed Interdigitated Electrodes. *Analyst* **2016**, *141*, 5441–5449, doi:10.1039/c6an00873a.
9. Güner, A.; Çevik, E.; Şenel, M.; Alpsoy, L. An Electrochemical Immunosensor for Sensitive Detection of Escherichia Coli O157:H7 by Using Chitosan, MWCNT, Polypyrrole with Gold Nanoparticles Hybrid Sensing Platform. *Food Chem* **2017**, *229*, 358–365, doi:10.1016/j.foodchem.2017.02.083.
